# Exploring the hereditary background of renal cancer in Denmark

**DOI:** 10.1371/journal.pone.0215725

**Published:** 2019-04-29

**Authors:** Maria Bejerholm Christensen, Karin Wadt, Uffe Birk Jensen, Charlotte Kvist Lautrup, Anders Bojesen, Lotte Nylandsted Krogh, Thomas van Overeem Hansen, Anne-Marie Gerdes

**Affiliations:** 1 Department of Clinical Genetics, Copenhagen University Hospital, Copenhagen, Denmark; 2 Department of Clinical Genetics, Aarhus University Hospital, Aarhus, Denmark; 3 Department of Clinical Genetics, Aalborg University Hospital, Aalborg, Denmark; 4 Department of Clinical Genetics, Sygehus Lillebaelt, Vejle, Denmark; 5 Department of Clinical Genetics, Odense University Hospital, Odense, Denmark; Ohio State University Wexner Medical Center, UNITED STATES

## Abstract

**Background:**

Every year more than 800 patients in Denmark are diagnosed with renal cell carcinoma (RCC) of which 3–5% are expected to be part of a hereditary renal cancer syndrome. We performed genetic screening of causative and putative RCC-genes (*VHL*, *FH*, *FLCN*, *MET*, *SDHB*, *BAP1*, *MITF*, *CDKN2B)* in RCC-patients suspected of a genetic predisposition.

**Methods:**

The cohort consisted of forty-eight Danish families or individuals with early onset RCC, a family history of RCC, a family history of RCC and melanoma or both RCC- and melanoma diagnosis in the same individual. DNA was extracted from peripheral blood samples or cancer-free formalin-fixed paraffin-embedded tissue.

**Results:**

One start codon variant of unknown clinical significance (VUS) (c.3G>A, p.Met1Ile) and one missense VUS (c.631A>C, p.Met211Leu) was found in *VHL* in a patient with RCC-onset at twenty-eight years of age but without other manifestations or family history of von Hippel-Lindau (VHL). Furthermore, in three families we found three different variants in *BAP1*, one of which was a novel non-segregating missense variant (c.1502G>A, p.Ser501Asn) in a family with two brothers affected with RCC. Finally, we found the known E318K-substitution in *MITF* in a RCC-affected member of a family with multiple melanomas. No variants were detected in *CDKN2B*.

**Conclusion:**

Although we did find three VUS’s in *BAP1* in three families and a pathogenic variant in *MITF* in one family, pathogenic germline variants in *BAP1*, *MITF* or *CDKN2B* are not frequent causes of hereditary renal cancer in Denmark. It is possible that the high prevalence of risk factors such as male gender, smoking and obesity has influenced the development of cancer in the patients of the current study. Further investigations into putative predisposing genes and risk factors of RCC are necessary to enable better prediction of renal cancer risk or presymptomatic testing of relatives in hereditary renal cancer families.

## Introduction

Renal cell carcinomas (RCCs) are the most frequent malignancies of the kidneys and comprise different subtypes with highly heterogeneous histopathology: 70–75% are clear cell (ccRCC), 10–16% are papillary (type 1 or 2), 5% are chromophobe, and the remaining 10% consists of other subtypes such as collecting duct and medullary tumors [1]. In Denmark, RCC represents 2–3% of all cancers with an average incidence of 825 cases/year for 2011–2015[2]. Cancer detection at an early stage with identification of small and localized tumors decreases morbidity and thus presymptomatic screening and surveillance of patients with high risk of RCC development is likely to decrease RCC mortality [3,4].

Twice as many men as women are diagnosed with RCC and a documented association between RCC and smoking, high blood pressure and obesity has been found. Several other risk factors for RCC such as environmental exposure to trichloroethylene (TCE) and cadmium, multiparity in women and pre-existing renal disease, e.g. acquired renal cysts, long term dialysis and kidney transplantation have been proposed, but the association has not been documented to the same degree [5].

Most cases of RCC are sporadic but occasionally familial clustering occurs. Early age of onset, multiple and/or bilateral lesions and several malignant and benign masses in the kidneys characterize RCC patients with a genetic predisposition [6]. Approximately 3–5% of RCCs are estimated to be part of a hereditary cancer syndrome of which most are inherited in an autosomal dominant fashion [7].

The majority of hereditary RCCs are caused by pathogenic germline variants in the *VHL* gene (OMIM #608537) that causes von Hippel-Lindau syndrome (VHL), while other predisposing syndromes include hereditary leiomyomatosis and RCC (*FH*, *OMIM#136850)*, Birt-Hogg-Dubé (*FLCN*, *OMIM #607273)*, hereditary papillary RCC *(MET*, *OMIM#164860)*, hereditary paraganglioma and RCC (*SDHB*, *OMIM#185470)* and constitutional chromosome 3 translocations of t(3;8)(p14.2;q24.1) (reviewed in [8], OMIM#14470). However, for several multi-case or early onset RCC families, screening for known pathogenic variants in the most frequent causative genes *VHL*, *FH*, *FLCN*, and *MET* yields no eligible explanation for the accumulation of RCC in the family, suggesting that unknown genes predisposing for RCC most likely exist.

One putative RCC-susceptibility gene is *BAP1*(OMIM#603089); a deubiquitinase associated with multiprotein complexes regulating pathways in the cell cycle, cellular differentiation and the DNA damage response [9]. *BAP1* is localized on the short arm of chromosome 3. A broad tumor spectrum accompanies *BAP1* pathogenic germline variants [10,11] and although this spectrum has not yet been fully elucidated, pathogenic variants in *BAP1* is known to predispose to cutaneous and uveal melanoma and mesothelioma and is suspected of playing a role in the development of other cancers such as breast cancer, cholangiocarcinoma [12], cancer of the pancreas [10] and basal cell carcinoma [13,14]. Furthermore *BAP1* has been found to be mutated in tissue from sporadic malignant renal tumors, which are associated with a high tumor grade and bad prognosis [15,16]. Pathogenic germline variants have been found to segregate with the disease in high risk RCC families in France and the US [17,18], indicating that RCC might be an integral part of the *BAP1* tumor spectrum.

This hypothesis has recently been supported by Haugh et al who by review of the literature reported twenty cases of RCC in 215 patients with pathogenic *BAP1*-variants corresponding to an estimated penetrance for RCC of 9% [19].Microphthalmia-associated transcription factor (MITF) regulates the expression of genes involved in the cell cycle, cellular proliferation and differentiation [20]. In 2011 Bertolotto et al discovered a germline amino acid substitution (E318K) in *MITF* (OMIM#156845) that occurred with a significantly higher frequency in patients with melanoma, RCC or both cancers when compared to controls [21]. The E318K variant has been found to increase growth, proliferation and migration of melanocytes and renal cells, and although the full picture of MITFs contribution to oncogenesis is yet unknown, the variant is thought to enable MITF to act as an oncogene and thereby predispose to melanoma and RCC [22].

*CDKN2B* (OMIM#600431) is located close to the tumor suppressor gene *CDKN2A* in region 9p21.3 on the short arm of chromosome 9; a region that often contains genetic alterations and is involved in the development of several types of tumors. CDKN2B controls cellular growth by forming complexes with CDK4 and CDK6, thus inhibiting progression into the G1 phase of the cell cycle [23]. *CDKN2B* has previously been linked to a wide variety of diseases including type-2 diabetes mellitus, atherosclerosis, primary open-angle glaucoma and leukemia. In 2015, Jafri et al discovered a novel nonsense variant in *CDKN2B* in a high risk RCC family. DNA was available from five family members of whom the variant was found in two affected brothers and not in three of their cancer free siblings. Furthermore, two novel missense variants in *CDKN2B* were found in a cohort of fifty individuals with features of nonsyndromic hereditary RCC [24].

In this study we have examined whether pathogenic variants in *BAP1*, *MITF* or *CDKN2B* play a role in the development of RCC. In Denmark, patients with early onset RCC and families with accumulation of RCCs are usually screened for variants in four genes: *VHL*, *FH*, *FLCN* and *MET*. At Copenhagen University Hospital *SDHB*-screening is also performed, since pathogenic variants in *SDHB* are correlated with an increased risk of paraganglioma, pheochromocytoma and RCC and standard chromosome analysis is performed to examine for the known chromosome translocation t(3;8)(p14.2;q24.1). We have performed analyses of the aforementioned RCC causative genes and screened for variants in the putative RCC genes *BAP1*, *MITF* and *CDKN2B*. Chromosome analysis has not been performed in the current study. We have analyzed DNA extracted from peripheral blood samples in forty-four families and DNA extracted from fresh-frozen paraffin embedded tissue in four families to further elucidate the genetic etiology of RCC and hopefully enable presymptomatic genetic testing to relatives in these families.

## Methods

### Study population

The study is conducted at the Department of Clinical Genetics, Copenhagen University Hospital, Copenhagen, Denmark in close collaboration with centers in Odense, Vejle, Aarhus and Aalborg, comprising all Departments of Clinical Genetics in Denmark. To be included in the study, patients must meet at least one of the criteria: RCC < forty years of age *or* bilateral or multifocal RCC < sixty years of age *or* family history of RCC *or* three relatives with RCC, melanoma or mesothelioma (at least one case of RCC) *or* patients diagnosed with RCC *and* melanoma. Family history of RCC is defined as two or more first-degree relatives with RCC. Patients with a previously identified pathogenic variant in a renal cancer predisposition gene were not included. Patient inclusion took place from February 2014 to January 2015.

**Fig 1:**
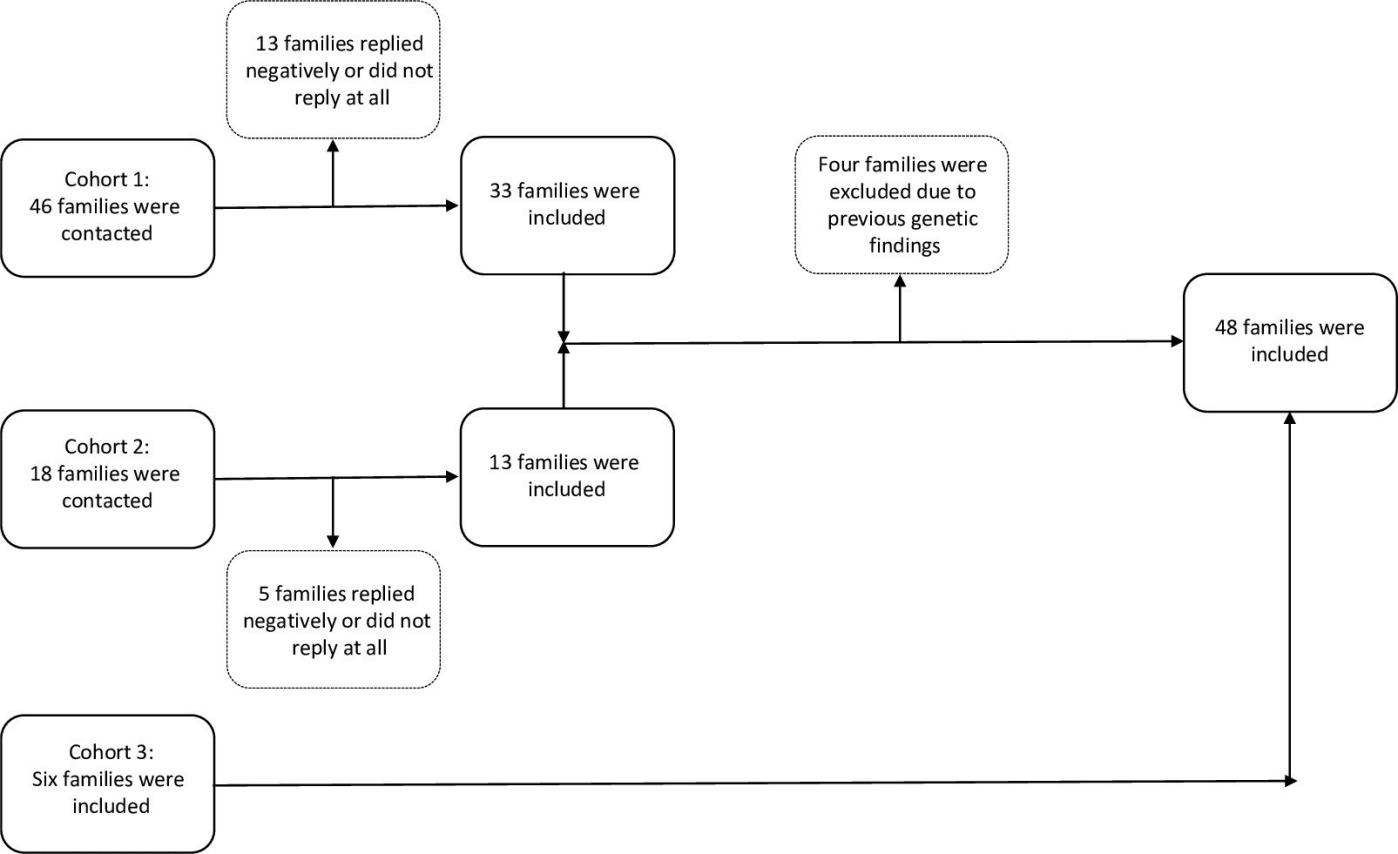
Flow chart illustrating the inclusion of patients. Cohort 1 comprises families previously genetically counselled. Cohort 2 comprises patients identified through the Danish Cancer Registry. Cohort 3 comprises one family identified through the clinical setting at Copenhagen University Hospital and five families identified from another study in the research group.

A total of sixty-four families were contacted. Eighteen families did not reply to our requests or were not interested in being a part of the study. Forty-six families accepted to be included, but after histopathological examination of the RCCs and examination of the proband’s medical history, four families were excluded due to having molecular genetically verified VHL (n = 2) or the renal tumor being a Wilms tumor (n = 2). Six families were included from another research study or clinical setting at Copenhagen University Hospital (Fig 1).

In six families, the proband was deceased and analyses were performed on cancer-free formalin-fixed paraffin-embedded tissue or DNA from banking of a peripheral blood sample taken at an earlier time.

As shown in Fig 1, the study population consists of three cohorts. Cohort 1 comprises thirty-two families, who had already been through genetic counselling and hence been screened for variants in one or more of the RCC predisposition genes *VHL*, *FH*, *FLCN* and *MET* without identification of any pathogenic mutations. One patient was identified through a keyword-based search of the medical record system used at Department of Clinical Genetics, Copenhagen University Hospital. Cohort 2 comprises nine families, who were included after identification through the Danish Cancer Registry. The Danish Cancer Registry contains information regarding all reported cancer incidents in Denmark since 1943. We received data regarding all patients in Denmark diagnosed with RCC before the age of forty *or* RCC at any age in addition to cutaneous or uveal melanoma or mesothelioma. Disease entities were denoted using ICD7 and ICD10. The oldest entry included in this study dated back to 1979. Cohort 3 comprises one family identified through the clinical setting at Copenhagen University Hospital and five families, who were identified and included from another study in the research group. These families have been described in the article ‘Molecular characterization of melanoma cases in Denmark suspected of genetic predisposition’ by Wadt et al [25]. These patients only consented to screening for variants in *BAP1* and the known E318K-variant in *MITF*, which was performed prior to this study. The study population consisted primarily of participants of European ethnicity and is therefore comparable to the participants of the studies referred to in this article. S1 Table contains detailed family and clinical information and data of genetic testing.

**Fig 2:**
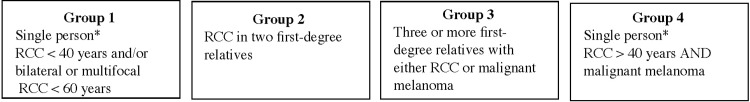
Schematic presentation of the organization of the participating families into four groups. The organization is based on clinical data. In group 1 one family member was affected with RCC *(n = 20)* either RCC < 40 years of age and/or bilateral or multifocal RCC < 60 years of age, in group 2 two family members were affected with RCC *(n = 21)*, in group 3 more than two family members were affected with RCC and/or melanoma (minimum of one RCC) *(n = 17)* and in group 4 the proband had been diagnosed with both melanoma *and* RCC *(n = 5)* comprising sixty-three family members in forty-eight families. *No affected first-degree relatives with RCC or malignant melanoma.

Once included all participating families were organized into four groups; in group 1 one family member was affected with RCC *(n = 20)*, in group 2 two family members were affected with RCC *(n = 21)*, in group 3 more than two family members were affected with RCC and/or melanoma (minimum of one RCC) *(n = 17)* and in group 4 the proband had been diagnosed with both melanoma *and* RCC *(n = 5)* comprising sixty-three family members in forty-eight families (Table 1 and Fig 2). A questionnaire was filled out regarding all RCC-affected family members possible to create a baseline description of the participants, and to collect data on risk factors for RCC and specific tumor details. Data for this baseline description and the exposure to risk factors of RCC was obtained through telephonic interviews with the participants.

**Table 1:** Probands and family members diagnosed with RCC.

	Group 1	Group 2	Group 3	Group 4	Average	Total
**Patients/tumors**	n = 20/25	n = 21/21	n = 17/18	n = 5/5		n = 63/69
**Average age at RCC-onset**	34.4	56.1	67.7	60.8	56.3	
**(Range)**	(25–57)	(34–73)	(51–89)	(43–69)	(25–89)	
**Average age at melanoma-onset**	-	-	49.7	48	49.3	
**(Range)**			(25–75)	(31–61)	(25–75)	
**Male gender**	65%	67%	53%	40%	59%	
**Smoking status**						
• **Non-smoker**	40%	29%	6%	20%	28%	
• **Smoker^a^**	40%	24%	29%	40%	38%	
• **Unknown**	20%	47%	65%	40%	34%	
**BMI**						
• **Average**	28	29	26	30	28	
• **Median**	27	31	25	29	28	
• **(Range)**	(20–39)	(21–35)	(21–34)	(29–33)	(20–39)	
• **Unknown**	n = 3	n = 10	n = 11	n = 2	n = 26	
**Histology**						
• **Clear cell**	13	14	10	1		38
• **Papillary**	0	1	4	0		5
• **Chromophobe**	2	0	0	1		3
• **Unknown**	9	5	4	3		21
• **Mixed^b^**	1	1	0	0		2

Group 1 comprises families with 1 case of RCC and an age of onset of 40 (unilateral) - 60 years (bilateral/multifocal). Group 2 comprises families with two cases of RCC. Group 3 comprises families with three or more cases of RCC and/or melanoma. Group 4 comprises patients diagnosed with both RCC and melanoma.

^a^’Smoker’ is defined as having smoked 100 cigarettes in a lifetime or having smoked daily for ≥6 months.

^b^Histological examination resulted in mixed histology consisting of clear cell + papillary + unknown histology in one tumor in group 1 and clear cell + papillary histology in one tumor in group 2.

If the RCC-affected family member was deceased, the questionnaire was filled out by the closest living relative on his or her behalf. This was done to the best of their knowledge, and when relevant data on a given parameter was impossible to obtain, the entity would be filled out as ‘unknown’. An extraction of the data can be seen in Table 1.

The patients were identified, and information obtained using the Danish Civil Registration System, in which all Danish citizens alive in 1968 or born hereafter, have been registered. All contacts to the Danish health care system, e.g. medical records, surgical descriptions, histopathological investigations etc. are linked to this registration. We used the Danish Pathology Data Bank to validate all cases of RCC and melanoma.

### Samples and DNA extraction

Thirty-three families included in this study have previously received genetic counseling at one of the five departments of clinical genetics in Denmark. Genetic counseling includes drawing a pedigree of at least three generations, validation of reported cancer cases and other relevant disease entities and collection of blood samples from the proband and/or relevant family members for genetic testing. In most families, surplus DNA samples from previous genetic tests were available for the current study. In the remaining families, the proband and relevant family members were asked to supply a peripheral blood sample. Basic genetic screening in patients with potential hereditary renal cancer in Denmark includes the four RCC-genes *VHL*, *FH*, *FLCN* and *MET*. The families included from another project in the research group (cohort 3) only consented to *BAP1*- and *MITF-*screening, but the basic screening was performed in all consenting cohort 1- and 2-families, had it not already been performed at the initial genetic counselling. Table 2 lists an overview of the genetic tests performed in the study, including the supplemented basic screening, on patients divided by group and S1 Table lists a detailed overview of genetic tests performed on each patient. Previously performed screenings by the clinical genetic departments were not repeated. When possible, we included *SDHB*, which is considered part of the basic screening at Copenhagen University Hospital. In this way we ensured that no pathogenic or likely pathogenic variants were present in any participant, who consented to basic genetic screening in the *VHL-*, *FH-*, *FLCN-* and *MET-* genes. In six families, the patient(s) diagnosed with RCC were deceased. In four of these families we sequenced the *VHL*, *FH*, *FLCN*, *MET*, *BAP1*, *MITF* and *CDKN2B*-genes using cancer-free formalin-fixed paraffin-embedded tissue from the autopsy or a previous operation. In two families, DNA from previous banking of a blood sample was available, however one unfortunately only held enough DNA for *CDKN2B*-analysis.

**Table 2:** Germline variant testing divided by group.

	Group 1	Group 2^a^	Group 3	Group 4	Total^a^
	tested/abnormal	tested/abnormal	tested/abnormal	tested/abnormal	tested/abnormal
***VHL***	17/1	14/0	5/0	2/0	38/1
***FH***	17/0	14/0	5/0	2/0	38/0
***FLCN***	17/0	14/0	5/0	3/0	39/0
***MET***	17/0	14/0	5/0	2/0	38/0
***SDHB***	9/0	7/0	1/0	0/0	17/0
***BAP1***	20/2	14/1^a^	8/0^b^	5/0	47/3
***MITF***	20/0	15/0^a^	6/1^b^^c^	5/0	46/1
***CDKN2B***	20/0	14/0^a^	5/0^d^	4/0^d^	43/0
**FFPE:Blood**	1:19	3:12	0:9	0:5	4:45

Table displaying the germline variant testing in the current study divided by group. For each gene the number in front of the fraction slash in denotes the number of patients tested. The number behind the fraction slash denotes the number of patients tested with abnormal results. The bottom row indicates the material tested in each group. Abbreviations: FFPE, formalin-fixed paraffin-embedded tissue.

^a^In one family both siblings diagnosed with RCC were screened for the E318K-variant in *MITF*, only one was screened for variants in *BAP1* and *CDKN2B*.

^b^In one family a previously banked blood sample only held enough DNA for *CDKN2B*-analysis.

^c^In two families a previously banked blood sample only held enough DNA for *BAP1*- and *CDKN2B*-analyses. The proband in one family had passed away prior to the present study and the proband in the other family passed away during the study period.

^d^The five families included from another project in the research group had only consented to *BAP1*- and *MITF*-analyses.

### Sequencing and MLPA analysis

Targeted next-generation sequencing (NGS) was performed from 2014 using a library designed to capture all exons from the *FH*, *FLCN*, *MET*, *SDHB* and *VHL* genes. Sequencing was performed on a MiSeq (Illumina) to an average depth of at least 100×. Sequencing data (FASTQ files) were analyzed using Sequence Pilot SeqNext software (v4.0 JSI medical systems), where variants were called if the non-reference base frequency was above 25%. The coding sequences of *BAP1* and *CDKN2B* (and *FH*, *FLCN*, *MET*, *SDHB* and *VHL* genes up to 2014) were amplified by PCR using primer pairs flanking each exon followed by sequencing on an ABI3730 DNA analyzer (Applied Biosystems). *MITF* was screened for the E318K variant by TaqMan analysis as recently described [25]. Moreover, genomic DNA was examined for large genomic rearrangements in the *FH*, *FLCN*, *SDHB*, and *VHL* genes by multiplex ligation-dependent probe amplification (MLPA) analysis using SALSA MLPA kits from MRC-Holland. *BAP1*, *MITF* and *VHL* variants are numbered according to accession number NM_004656.3, NM_000248.3 and NM_000551.3, respectively, following the guidelines from the Human Genome Variation Society (http://www.hgvs.org/varnomen). These techniques are not able to detect large rearrangements.

Variants were classified as ‘pathogenic’ (class 5), ‘likely pathogenic’ (class 4), ‘variants of unknown significance’ (class 3), ‘likely benign’ (class 2) or ‘benign’ (class 1) following the IARC classification system [26]. Variants classified as ‘pathogenic’ or ‘likely pathogenic’ are defined as having a more than 0.99 or 0.95–0.99 probability of actually being pathogenic, respectively, whereas variants classified as a having ‘unknown significance’ have 0.05–0.949 probability of actually being pathogenic and should not be used for predictive testing. ‘Likely benign’ and ‘benign’ variants have a low probability of actually being pathogenic (0.001–0.049 and lower than 0.001, respectively) and are treated as ‘no mutation detected’ for RCC.

### Ethics

Collection of data regarding the patients’ medical history and initiation of genetic analyses were performed after written consent had been obtained. We only contacted individuals older than 18 years of age. Potential participants were contacted by letter with information regarding the study. If interested in participating, patients were informed about the project by phone and were sent a letter of consent to sign. When the signed consent form was received, patients were contacted by phone to obtain medical and family history, to fill out a questionnaire regarding risk factors of renal cancer and to be given the opportunity to ask any questions there might be regarding the study. Participants have filled out a written consent form to the use of pedigrees in this article.

The participants were included in a password protected database with name, CPR-number, data regarding their renal tumor(s) and risk factor questionnaire, and was given a constructed family number. Only MBC and KW had access to this database. To ensure accuracy, blood samples were marked with CPR-number, while extracted DNA was anonymized.

The study was approved by the Danish Data Protection Agency and the Committee on Health Research Ethics in the Capital Region of Denmark (H-1-2013-129).

## Results

### Variants

**Fig 3:**
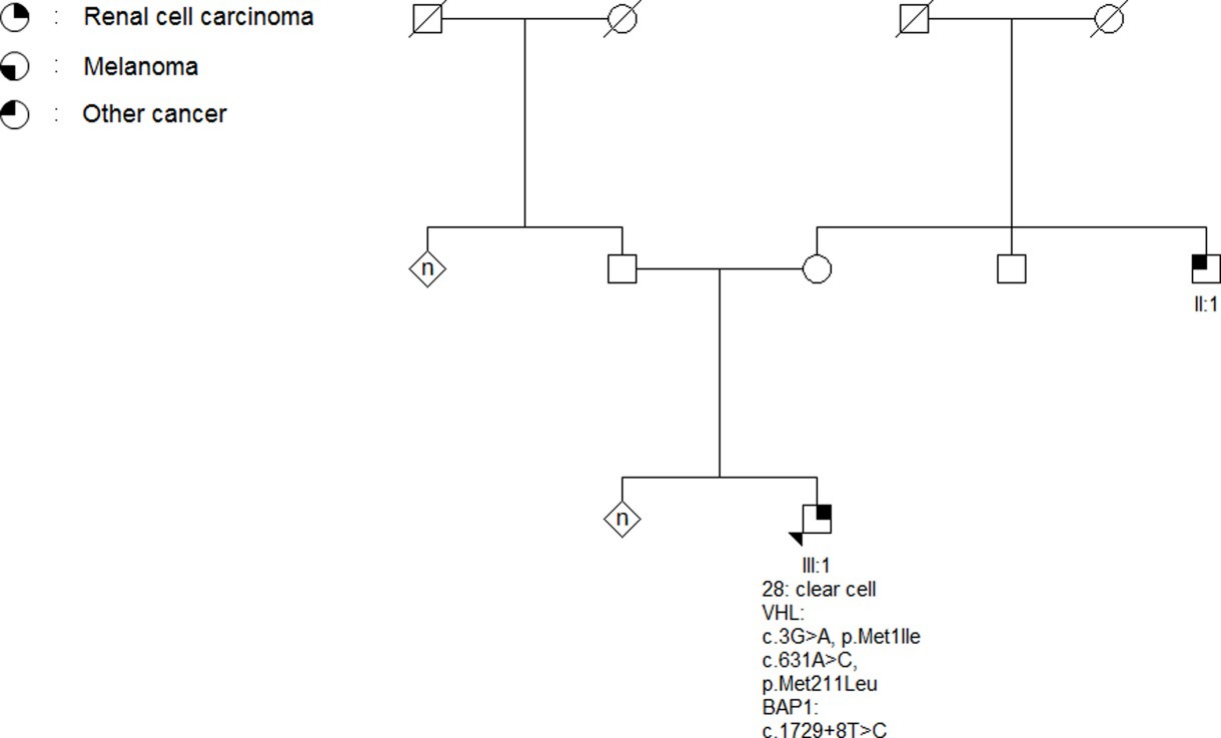
Pedigree of family 6002. The arrow indicates the proband. The age of RCC-onset and histological subtype the renal tumor is listed below the proband (III:3). A start codon VUS (c.3G>A, p.Met1Ile) and a missense variant (c.631A>C, p. Met211Leu) in *VHL* and a intron variant (c.1729+8T>C) in *BAP1* was identified in a peripheral blood sample from the proband. The proband’s maternal uncle (II:1) was diagnosed with testicular cancer.

Sixteen patients were screened for variants in one or more of the RCC predisposition genes *VHL*, *FH*, *FLCN*, *MET* or *SDHB* (Table 2). No pathogenic variants were found in *FH*, *FLCN*, *MET* or *SDHB*, but we did find a start codon variant (c.3G>A, p.Met1Ile) and a missense variant (c.631A>C, p.Met211Leu) in *VHL* in a patient from cohort 2 (Fig 3). Both *VHL* variants are classified as class 3 variants (VUS) according to the classification guidelines from IARC [26].

**Fig 4:**
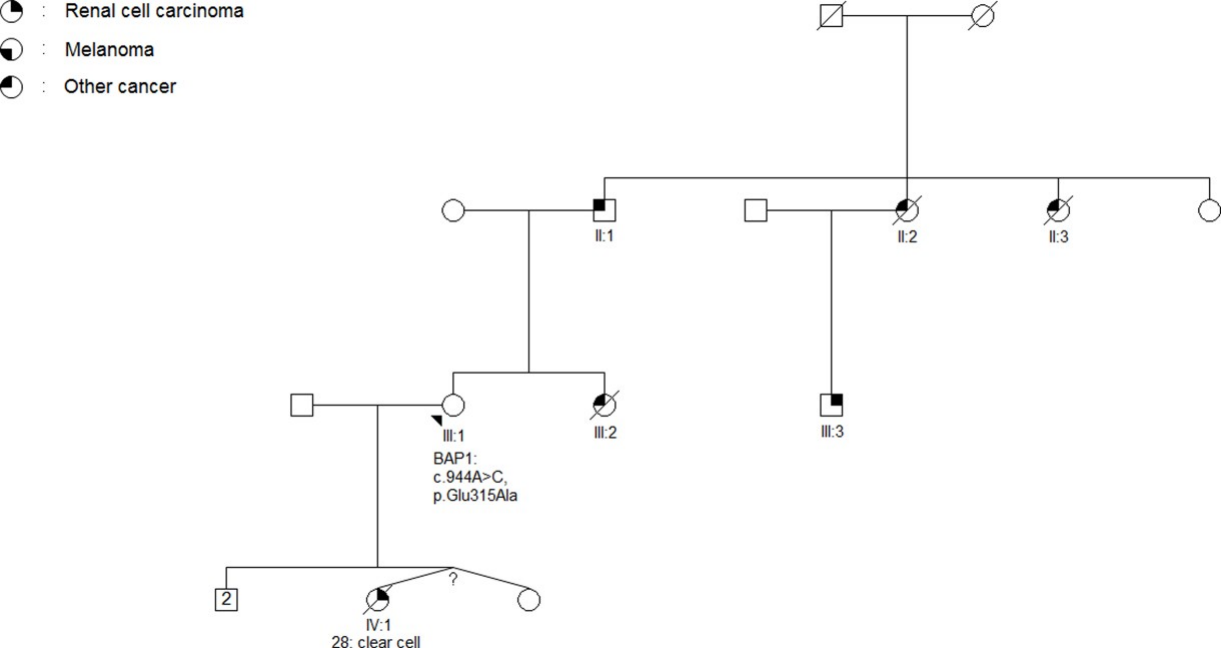
Pedigree of family 5001. The arrow indicates the proband. The age of RCC-onset and histological subtype of the primary renal tumor is listed below the patient (IV:1). A VUS (c.944A>C, p.Glu315Ala) in *BAP1* was identified in a peripheral blood sample from the proband. According to the family’s wishes we did not contact III:3. Additional cancers in the family are as follows: II:1: Prolactinoma, II:2: Pancreas, II:3: Thyroid, III:2 Sarcoma.

**Fig 5:**
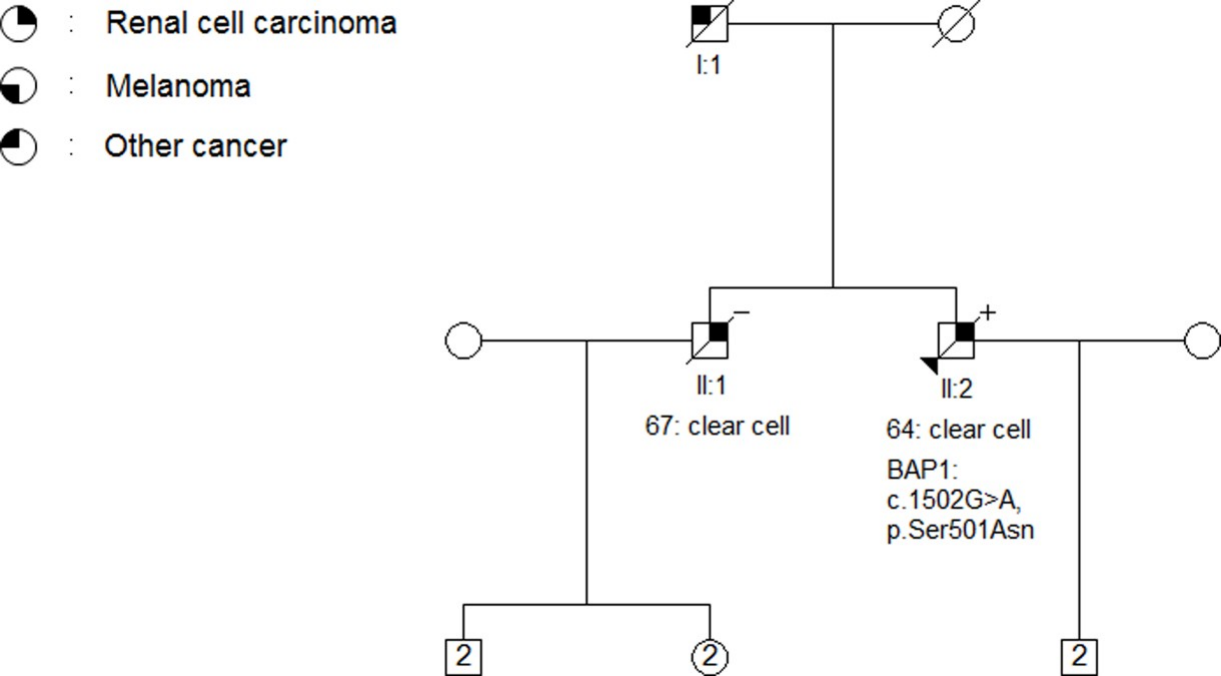
Pedigree of family 1013. The arrow indicates the proband. The age of RCC-onset and histological subtype of each primary renal tumor is listed below the patient. A novel VUS (c.1502G>A, p.Ser501Asn) in *BAP1* was identified in a peripheral blood sample from the proband (+) but was not found in two samples of FFPE from the proband’s brother (-).The proband’s father (I:1) was diagnosed with lung cancer.

In three patients of forty-seven tested, we found variants in *BAP1*; two missense variants (c.944A>C, p.Glu315Ala, Fig 4) and (c.1502G>A, p.Ser501Asn, Fig 5) and one intron variant (c.1729+8T>C, Table 3). The missense variant c.944A>C, p.Glu315Ala is reported in the gnomAD database with a frequency of 0.016% in Europeans (non-Finnish). We classify the variant as a class 3 variant.

**Table 3:** Variants found in the present study and clinical characteristics of the families.

ID	Age of RCC- onset	Gene	Variant type	Coding DNA variant	Protein variant	Population frequency (gnomAD NFE)	Clinical significance	Other cancers in the family
6002, III:1	28	VHL	Start codon	c.3G>A	p.Met1Ile	0.0016%	VUS	Testis
		VHL	Missense	c.631A>C	p.Met211Leu	0.00079%	VUS	
		BAP1	Intron variant	c.1729+8T>C		0.60%	VUS	
5001, III:1	28	BAP1	Missense	c.944A>C	p.Glu315Ala	0.016%	VUS	Sarcoma, prolactinoma, thyroid, pancreas
1013, II:2	65	BAP1	Missense	c.1502G>A	p.Ser501Asn	NR	VUS	Lung
7002	80	MITF	Missense	c.952G>A	p.Glu318Lys	0.25%	Pathogenic	Basal cell carcinoma, squamous cell carcinoma, melanoma, breast

The intron variant c.1729+8T>C (rs150945583), which is identified in the patient with two *VHL* variants, is reported in the gnomAD database with a frequency of 0.60% in Europeans (non-Finnish). *In silico* splicing analysis indicates that the variant has no effect on splicing and hence it is classified as a class 2 variant. Finally, the novel missense variant c.1502G>A, p.Ser501Asn is not reported in the gnomAD database and to our knowledge not in the literature, and is classified as a class 3 variant. Sequencing for the E318K-variant in *MITF* was performed in forty-five patients with normal results. In one family, we were able to collect DNA from two relatives with RCC and both were screened for the E318K variant with normal results. In one family, the patient diagnosed with RCC was found to carry the E318K variant. The patient was also diagnosed with two non-melanoma skin cancers. Two daughters were diagnosed with melanoma, one of whom was a carrier of the E318K-variant. The family has been further described by Wadt et al [25].

The screening of *CDKN2B* in forty-three patients revealed no variations. An overview of the genetic tests performed, and variants found, can be seen in Tables 2 and 3 and S1 Table.

### Non-genetic risk factors

The average age of RCC-onset in group 1 was 34.4 years, which is more than twenty years younger than the normal age range of onset of RCC in Denmark (60–69 years). This is most likely a result of the inclusion criteria and the definition of group 1 and is therefore not surprising.

The age of melanoma onset in group 3 and 4 corresponds to the normal age range of melanoma onset in the Danish population. We see a surplus of men, smokers and obese in this study consistent with the known risk factors for RCC. Two patients could not rule out having worked with TCE or cadmium, but no certain exposure was documented. No patients had acquired renal cysts, been through renal dialysis or had a renal transplantation prior to the onset of RCC.

### Histology

As expected, the ccRCC was by far the most common histologic subtype. Of the seven papillary RCCs, two were found in the same family (II:1 and II:2, Fig 6) in which five family members had been diagnosed with RCC, one with two primary tumors. The remaining four tumors in the family were ccRCC (II:3, III:1 x 2 and III:2).

**Fig 6:**
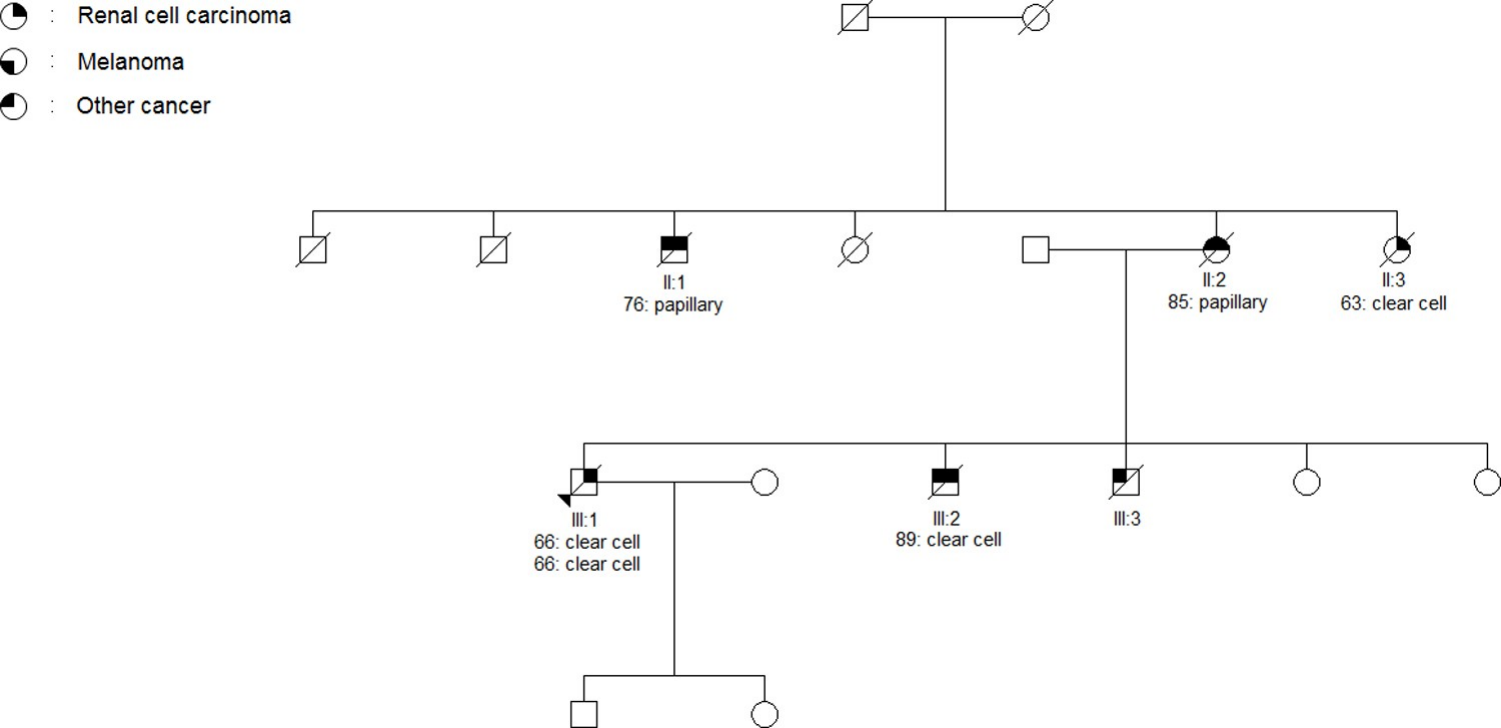
Pedigree of family 2006. The arrow indicates the proband. The age of RCC-onset and histological subtype of each primary renal tumor is listed below the patient. Additional cancers in the family are as follows: II:1: Lung, II:2: Rectum, III:2: Prostate, III:3: Unknown.

One patient with mixed histology (clear cell + papillary (+ unknown)) had a relative with ccRCC. The three chromophobe RCCs occur in three different families with RCC-onset of twenty-five years, thirty-two years and sixty-five years, respectively. We classified 30% of tumors as unknown histology since it was not possible to find a thorough histopathological description in the Danish Pathology Data Bank or medical records or because the pathologists were unable to classify the RCC tumor.

## Discussion

We found three VUS’s in *BAP1* and two VUS’s in *VHL*. Forty-six families in the current study, including thirteen families with melanoma, have been screened for the known E318K-variant in *MITF*, but the variant has only been found in one family, which has previously been published [25]. No variants were found in *CDKN2B*. Our cohort was characterized by a surplus of men, and most patients were overweight and exposed to smoking, all of which are known risk factors for RCC.

### BAP1

The c.1502G>A, p.Ser501Asn variant in *BAP1* in family 1013 has not previously been reported. Pathogenic clues include the highly conserved nucleotide and amino acid involved, and a SIFT and MutationTaster prediction as ‘deleterious’. However, the variant was not found in two cancer-free FFPE samples collected from the proband’s brother also diagnosed with RCC (II:1, Fig 5) and thus did not segregate with RCC in the family. No distant relatives in the family are known to have been diagnosed with RCC, and therefore it is not possible to perform further segregation analyses. The variant is classified as a VUS (class 3).

The c.944A>C, p.Glu315Ala variant in *BAP1* in family 5001 has not been reported in families with *BAP1*-associated cancers but has a population frequency of 0.016%. Like the above-mentioned variant, this variant also includes highly conserved amino acids. The physicochemical difference between glutamic acid and alanine is however moderate, and in silico analyses does not provide a clear answer as to whether the variant is pathogenic or benign. Thus, the variant is classified as a VUS (class 3).

### VHL

The start codon variant (c.3G>A, p.Met1Ile) and missense variant (c.631A>C, p.Met211Leu) in VHL in family 6002 was found in a patient (III:1, Fig 3), who was diagnosed with ccRCC at age twenty-eight, but neither he nor any family member have other VHL-manifestations. The start codon variant substitutes the initial methionine, thus skipping the native start codon with a yet unknown effect on VHL translation. An alternative start codon at codon 54, which harbors the next methionine, is presumed to initiate VHL translation, thus forming an alternative protein product, which is thought to possess tumor suppressor properties partially similar to wildtype VHL [27–30]. The p.Met211Leu variant is weakly conserved and there is a small physiochemical difference between methionine and leucine, indicating that the variant is benign. However due to the lack of functional data, both variants are classified as VUS’s. It is possible that mosaicism of a third, and pathogenic, variant in VHL could explain the mild VHL-phenotype. The patient has been through the national screening program for putative VHL, which includes MRI of the cerebrum, spinal medulla and abdomen, neurological examination, auditory tests, ocular examination and blood work for chromogranin A and metanephrines, with normal results. No nationally accepted surveillance program for early renal cancer exits, but due to his young age at RCC-onset, the patient and his first-degree relatives have been offered renal ultrasounds every two years.

### MITF

Evidence suggests that the E318K-variant plays a role in the development of melanoma but the role in the development of RCC is unclear. Recent studies do not find an association between the E318K-variant and the development of RCC, neither does the current study [31,32]. A possible association between the E318K-variant and the development of pheochromocytoma and paraganglioma has been proposed recently by Castro-Vega et al, illustrating that the cancer spectrum of the E318K-variant, if any, is not yet fully elucidated [33]. Further studies should be performed in renal cancer families to elucidate the impact of the E318K-variant in the development of RCC.

### CDKN2B

We found no variants in *CDKN2B* and are therefore not able to confirm the findings of Jafri et al regarding *CDKN2B* as a RCC predisposing gene [24].

Two different putative RCC-genes include *PTEN* known for its involvement in Cowden syndrome, in which patients have a genetic predisposition to RCC, and *PBRM1* in which a heterozygote germline variant was found to segregate with RCC in four affected family members in a French RCC-family [34]. The proband of one family in the current study was screened for variants in *PTEN* prior to inclusion, but in the remaining families *PTEN* screening was not considered relevant by the Departments of Clinical Genetics initially performing the genetic counselling. The families in cohort 2 and 3 did not have pedigrees suggestive of Cowden syndrome.

We have not investigated the involvement of *PBRM1* in RCC-tumorigenesis. Our cohort consisted of forty-eight families suspected of having a genetic predisposition to RCC, but where the most common RCC-associated syndromes had been excluded. The average age of RCC-onset in this study is 56.3 years (Table 1), which is younger than the average onset of RCC in the Danish population (64.4 years in 2013). Excluding group one, which is heavily biased in this aspect, the average age of RCC-onset in this study is 61.3 years, only 3.1 years younger than the average onset in the general population. Early onset of RCC could indicate genetic susceptibility to cancer, but in regard to other life style factors known to be risk factors of RCC, the patients in this study have a high consumption of tobacco and are generally obese. In 2017 the National Board of Health published ‘Danskernes Sundhed—Den Nationale Sundhedsprofil’, a report created using data from questionnaires filled out by more than 170,000 Danish citizens [35], 16.9% of responders report that they smoke daily. The highest percentage of the population smoking daily is in the age group 55–64 years, in which 21.9% of men and 20.3% of women report daily smoking. In the age group 16–44 years, 17.1%-18.4% (men) and 13.5–15.3% (women) of the population report daily smoking. In the current study a large percentage of the patients in each group, 40% of the patients in group 1 and 4, are reported as smokers, which is massive, compared to the general population, albeit the sample size is small. The average BMI is >25 in all groups and in group 4 all participants are overweight. In the general population 57.7% of men and 44.4% has a BMI of >25. Only four patients in this study have a BMI <25 and are non-smokers. The average age on RCC-onset in these patients is 47.6 years (28–66 years). We see a surplus of men in the study except for group 4, where the surplus of women might reflect that more women than men expose themselves to high amounts of UV-radiation leading to development of melanoma.

Since habits such as smoking, sedentary lifestyle and unhealthy diet tends to accumulate in families as well as genetic predisposition, distinction of the influence of hereditary and environmental factors on RCC is difficult. The most likely explanation to the RCC diagnoses in the current study is that many cases of RCC are multifactorial; arising from a combination of moderate genetic risk factors for RCC and non-genetic risk factors and exposure to environmental factors might play a non-negligible role in the development of renal cancer in some of the individuals of the current study.

Of the tumors in this study 79% were described as clear cell, 10% were papillary and 6% chromophobe consistent with the distribution found in the literature. It was not possible to classify 30% of tumors. Histopathological sub classifications of RCCs are done by evaluating the morphology of the tumor specimen. Until 2016, the ‘2004 WHO Classification of the Renal Tumors of the Adults’ has been used as a reference for the morphological description. Here ten different histological subtypes of RCCs are described (clear cell, multilocular cystic, papillary, chromophobe, collecting duct carcinoma, medullary carcinoma, RCC associated with Xp11.2 translocations, RCC associated with neuroblastoma, mucinous, tubular and spindle cell carcinomas and the unclassified carcinomas) [36]. Immunohistochemistry is recommended to elucidate or support the classification if the morphology is unclear. In their article from 2011 Algaba et al describes further sub classifications of RCC including the subtype clear cell papillary RCC, with morphological changes from both subtypes in one tumor [37] and in the new '2016 WHO Classification of the Renal Tumors of the Adults' the ‘clear cell papillary renal cell carcinoma’ has been denoted as a separate entity [38]. It is possible, that the two mixed tumors included in this study, should in fact be reclassified as belonging to this subgroup. Clinical relevance of the new classification is not yet evident. Two *BAP1-*variant carriers (6002, III:1 and 1013, II:2 (Table 3)) was diagnosed with ccRCC and one *MITF*-variant carrier (7002, (Table 3)) was diagnosed with papillary RCC. Few E318K-carriers have been diagnosed with RCC, but the most prevalent histological subtype described in the literature is clear cell carcinoma [21]. One of the strengths of this study is its national approach comprising families previously genetically counselled due to suspected hereditary RCC syndrome. Seventy-two percent of the invited patients accepted to be included in the study. However, some families with several cases of RCC might not have been identified prior to this study and therefore have not been offered genetic counselling. Some have not been interested. To minimize this selection bias, we have applied data from the Danish Cancer Registry about all Danish citizens with an onset of RCC before the age of forty since 1979. These patients were denoted cohort 2, which also includes the patients with RCC and melanoma found through the Danish Cancer Registry. Throughout the study, we have identified and monitored patients through the Danish Civil Registration System and validated RCCs and other tumors of interest using the Danish Pathology Data Bank, giving the data in the study high validity.

### Conclusion

We screened forty-nine family members in forty-eight families for variants in one or more of the RCC causative genes *VHL (n = 10)*, *FH (n = 11)*, *FLCN (n = 10)*, *MET (n = 11)* and *SDHB (n = 9)* and the putative RCC-genes *BAP1 (n = 47)*, *MITF (n = 46)* and *CDKN2B (n = 43)*. We found two VUS’s in *VHL* in a patient with onset of RCC at twenty-eight years of age and no family history of RCC or other VHL-manifestations. Furthermore, we found three VUS’s in *BAP1*, of which one was novel and non-segregating in the family, and the E318K variant in *MITF* in one patient of a family with melanoma and RCC. Since the *BAP1* variants are classified as VUS’s, a standardized surveillance program for variant carriers is not relevant. A surveillance program has not been established for E318K variant carriers, but increased awareness of potentially malignant cutaneous lesions and symptoms of renal disease should be advised. In a clinical setting, we now routinely perform genetic testing of *VHL*, *MET*, *FH*, *FLCN*, *MITF*, *BAP1*, *PTEN*, *SDHB*, and *CDKN2B* in individuals and families suspected for genetic predisposition to RCC. The results of the study indicate that germline variants in *BAP1*, *MITF* and *CDKN2B* are not frequent in Danish families with suspected hereditary predisposition to renal cancer, and in the families of this study a possible genetic background of RCC is still unresolved. Furthermore, the results indicate that non-genetic risk factors of renal cancer play a key role in renal cancer pathogenesis in families suspected of hereditary predisposition to renal cancer where the most common RCC syndromes have been excluded. Further genetic, epigenetic and epidemiological investigations into putative predisposing genes and risk factors of RCC are necessary to fully elucidate RCC-pathogenesis and predict risk of renal cancer in families with hereditary predisposition to renal cancer.

## Supporting information

S1 Table Detailed family and clinical information and data regarding genetic testing. The table includes information of age of RCC, occurrence of RCC or malignant melanoma in the family, and which genes have been examined in a clinical setting, or in the project.
Abbreviations: Co: Cohort; RCC: age RCC, with multiple ages, multiple primary tumors; X: Genetic analysis has been performed within the project; g: prior genetic analysis (NGS or Sanger sequencing); * not in first-degree relative; ** two first-degree relative and one more distant relative; *** family report; RCC total: total affected with RCC in the family; MM total: Total affected with MM in the family; CM: cutaneous melanoma; UM: uveal melanoma; VAR: variant detected.
(DOCX)
