# Supplementary material for: Exploring the hereditary background of renal cancer in Denmark
Source: PLoS One. 2019 Apr 29;14(4):e0215725. doi: 10.1371/journal.pone.0215725 (PMC6488054; doi:10.1371/journal.pone.0215725)
Supplement: S1 Table — The table includes information of age of RCC, occurrence of RCC or malignant melanoma in the family, and which genes have been examined in a clinical setting, or in the project. Abbreviations: Co: Cohort; RCC: age RCC, with multiple ages, multiple primary tumors; X: Genetic analysis has been performed within the project; g: prior genetic analysis (NGS or Sanger sequencing); * not in first-degree relative; ** two first-degree relative and one more distant relative; *** family report; RCC total: total affected with RCC in the family; MM total: Total affected with MM in the family; CM: cutaneous melanoma; UM: uveal melanoma; VAR: variant detected. (DOCX) [file pone.0215725.s001.docx]

**S1 Table. Detailed family and clinical information and data regarding genetic testing**

| ID | Co | RCC | MM | RCC total | MM total | *VHL* | *FH* | *FLCN* | *MET* | *SDHB* | *BAP1* | *MITF* | *CDKN2B* |
| --- | --- | --- | --- | --- | --- | --- | --- | --- | --- | --- | --- | --- | --- |
| **GROUP 1** |  |  |  |  |  |  |  |  |  |  |  |  |  |
| 1001 | 1 | 53/53 | no | no | no | X | X | X | X | - | X | X | X |
| 1002 | 1 | 25 | no | no | no | g | g | g | g | g | X | X | X |
| 1005 | 1 | 33 | no | no | no | g | g | g | g | X | X | X | X |
| 1007 | 1 | 38/55 | no | no | no | g | g | g | g | g | X | X | X |
| 1008 | 1 | 38 | no | no | no | X | X | X | X | X | X | X | X |
| 1010 | 1 | 32 | no | no | no | g | g | g | g | g | X | X | X |
| 1011 | 1 | 32 | no | 3* | no | g | g | g | g | X | X | X | X |
| 1015 | 1 | 35 | no | no | no | g | g | g | g | g | X | X | X |
| 1018 | 1 | 39 | no | no | no | X | X | X | X | - | X | X | X |
| 2001 | 1 | 38 | no | no | no | g | g | g | g | - | X | X | X |
| 4001 | 1 | 29 | no | 2* | no | g | g | g | g | - | X | X | X |
| 4002 | 1 | 36/46 | no | no | no | g | g | g | g | - | X | X | X |
| 5001 | 1 | 28 | no | no | no | X | X | X | X | X | VAR | X | X |
| 5003 | 1 | 40/41/57 | no | no | no | g | g | g | g | - | X | X | X |
| 5004 | 1 | 42 | no | 2* | no | g | g | g | g | - | X | X | X |
| 6001 | 2 | 29 | no | no | no | X | X | X | X | X | X | X | X |
| 6002 | 2 | 28 | no | no | no | VAR | X | X | X | - | VAR | X | X |
| 6006 | 2 | 37 | no | no | yes | - | - | - | - | - | X | X | X |
| 6007 | 2 | 30 | no | no | no | - | - | - | - | - | X | X | X |
| 6008 | 2 | 32 | no | no | no | - | - | - | - | - | X | X | X |
| **GROUP 2** |  |  |  |  |  |  |  |  |  |  |  |  |  |
| 1003 | 1 | 68 | no | 3** | no | g | g | g | g | g | X | X | X |
| 1004 | 1 | 46 | no | 2 | no | X | X | X | X | - | X | X | X |
| 1006 | 1 | 59 | no | 3** | no | g | g | g | g | g | X | X | X |
| 1009 | 1 | 66 | no | 2 | no | g | g | g | g | g | X | X | X |
| 1012 | 1 | 58 | no | 2 | no | X | X | X | X | - | X | X | X |
| 1013 | 1 | 65 | no | 2 | no | X | X | X | X | - | VAR | X | X |
| 1014 | 1 | 34 | no | 2 | no | g | X | g | X | X | X | X | X |
| 2002-1 | 1 | 54 | CM | 2 | 1 | g | g | g | g | - | X | X | X |
| 2002-2 | 1 | 50 | no | 2 | 1 | - | - | - | - | - | - | X | - |
| 2003 | 1 | 73 | no | 3 | no | g | g | g | g | - | X | X | X |
| 2005 | 1 | 54 | no | 2 | no | g | g | g | g | X | X | X | X |
| 2008 | 1 | 49 | no | 2 | no | g | g | g | g | X | X | X | X |
| 2009 | 1 | 40 | no | 2 | no | X | X | X | X | - | X | X | X |
| 3002 | 1 | 57 | no | 3 | no | g | g | g | g | X | X | X | X |
| 5002 | 1 | 51 | no | 2 | no | g | g | g | g | - | X | X | X |
| **GROUP 3** |  |  |  |  |  |  |  |  |  |  |  |  |  |
| 2006 | 1 | 66/66 | no | 5 | no | g | g | g | g | - | - | - | X |
| 2007 | 1 | 56 | no | 2 | 2 | g | g | g | g | - | X | X | X |
| 3001 | 1 | 64 | CM | no | 2 | g | g | g | g | g | X | X | X |
| 5005 | 1 | 54 | no | 3 | no | g | g | g | g | - | X | - | X |
| 7001 | 3 | 51 | no | 3 | no | g | g | g | g | - | X | - | X |
| 7002 | 3 | 81 | CM | no | 3 | - | - | - | - | - | X | VAR | - |
| 7003 | 3 | 73 | no | no | 2 | - | - | - | - | - | X | X | - |
| 7005 | 3 | 61 | no | no | 2 | - | - | - | - | - | X | X | - |
| 7006 | 3 | 84 | no | no | 5 | - | - | - | - | - | X | X | - |
| **GROUP 4** |  |  |  |  |  |  |  |  |  |  |  |  |  |
| 6003 | 2 | 58 | CM | no | no | g | g | g | g | - | X | X | X |
| 6004 | 2 | 68 | UM | no | no | - | - | - | - | - | X | X | X |
| 6005 | 2 | 43 | CM | no | no | - | - | - | - | - | X | X | X |
| 6009 | 2 | 69 | UM | 1*** | no | g | g | g | g | - | X | X | X |
| 7004 | 3 | 65 | CM | no | no | - | - | g | - | - | X | X | - |

Abbreviations: Co: Cohort; RCC: age RCC, with multiple ages, multiple primary tumors; X: Genetic analysis has been performed within the project; g: prior genetic analysis (NGS or Sanger sequencing); * not in first-degree relative; ** two first-degree relative and one more distant relative; *** family report; RCC total: total affected with RCC in the family; MM total: Total affected with MM in the family; CM: cutaneous melanoma; UM: uveal melanoma; VAR: variant detected
